# Functionality and prevalence of trehalose-based oligosaccharides as novel compatible solutes in ascospores of *Neosartorya fischeri (Aspergillus fischeri)* and other fungi

**DOI:** 10.1111/1462-2920.12558

**Published:** 2014-10-22

**Authors:** Timon T Wyatt, M Richard van Leeuwen, Elena A Golovina, Folkert A Hoekstra, Eric J Kuenstner, Edward A Palumbo, Nicole L Snyder, Cobus Visagie, Alex Verkennis, John E Hallsworth, Han AB Wösten, Jan Dijksterhuis

**Affiliations:** 1 CBS-KNAW Fungal Biodiversity CentreUppsalalaan 8, Utrecht, 3584CT, The Netherlands; 2 Laboratory of Biophysics, Wageningen NMR Centre, Wageningen UniversityWageningen, The Netherlands; 3 Laboratory of Plant Physiology, Wageningen UniversityWageningen, The Netherlands; 4 Chemistry Department, Hamilton CollegeClinton, NY, USA; 5 Department of Chemistry, Davidson CollegeDavidson, NC, USA; 6 Institute for Global Food Security, School of Biological Sciences, Medical Biology Centre, Queen's University BelfastBelfast, Northern Ireland; 7 Microbiology and Kluyver Centre for Genomics of Industrial Fermentation, Institute of Biomembranes, Utrecht UniversityUtrecht, The Netherlands

## Abstract

Ascospores of *Neosartorya*, *Byssochlamys* and *Talaromyces* can be regarded as the most stress-resistant eukaryotic cells. They can survive exposure at temperatures as high as 85°C for 100 min or more. *Neosartorya fischeri* ascospores are more viscous and more resistant to the combined stress of heat and desiccation than the ascospores of *Talaromyces macrosporus* which contain predominantly trehalose. These ascospores contain trehalose-based oligosaccharides (TOS) that are novel compatible solutes, which are accumulated to high levels. These compounds are also found in other members of the genus *Neosartorya* and in some other genera within the order Eurotiales that also include *Byssochlamys* and *Talaromyces*. The presence of oligosaccharides was observed in species that had a relatively high growth temperature. TOS glasses have a higher glass transition temperature (T_g_) than trehalose, and they form a stable glass with crystallizing molecules, such as mannitol. Our data indicate that TOS are important for prolonged stabilization of cells against stress. The possible unique role of these solutes in protection against dry heat conditions is discussed.

## Introduction

Extremely stress-resistant ascospores are found among the fungal genera *Neosartorya*, *Byssochlamys* and *Talaromyces*. These spores resist high temperature, pressure and desiccation (Beuchat, 1986; Dijksterhuis and Samson,^2002^,^2006^; Reyns *et al*., 2003; Dijksterhuis and Teunissen, 2004; Dijksterhuis *et al*., 2007; Houbraken *et al*., 2012a) and are arguably the most stress-resistant eukaryotic cells described to date, with a temperature resistance similar to *Bacillus subtilis* spores (Dijksterhuis and Teunissen, 2004). Therefore, these ascospores can survive pasteurization and cause spoilage of food products (Tournas, 1994). The pasteurization treatment can even break the dormancy of these ascospores leading to germination (Reyns *et al*., 2003; Dijksterhuis and Teunissen, 2004; Dijksterhuis and Samson, 2006). Humidity is an important determinant for stress resistance. Ascospores of *Neosartorya fischeri* exposed to extreme heat (95°C) at a relative humidity (RH) of 30% had a D-value (decimal reduction time, or the time that is required to kill 90% of the spores) almost 200 times higher than spores exposed to a RH of 75% (Gomez *et al*., 1994).

Compatible solutes, including polyols, sugars, betaines and amino acids, protect cells against stresses such as desiccation and high temperature both of which impact the cellular system at the level of water : macromolecule interactions. These molecules are compatible with cellular functioning even when present at high concentration. The sugar trehalose and the polyols glycerol, erythritol, arabitol and mannitol are the major solutes in fungal cells. Glycerol is the predominant compatible solute that accumulates upon osmotic stress in many fungi (Redkar *et al*., 1995; Hagiwara *et al*., 2007; Kogej *et al*., 2007), but erythritol, arabitol or mannitol can also be found (Managbanag and Torzilli, 2002; Ruijter *et al*., 2004; Chin *et al*., 2010). Stress-resistant ascospores contain large amounts of trehalose and mannitol (Conner *et al*., 1987; Dijksterhuis and Samson, 2002; Dijksterhuis *et al*., 2002). For instance, *Talaromyces macrosporus* ascospores accumulate trehalose at up to 17% of the fresh weight, in addition to mannitol (Dijksterhuis and Samson, 2002; Dijksterhuis *et al*., 2002). Trehalose and mannitol are also the most abundant solutes in conidia (asexual spores) when grown on high water activity and nutrient-rich media (Tereshina *et al*., 2000,2004; Fillinger *et al*., 2001; Ruijter *et al*., 2003; Doehlemann *et al*., 2006; Solomon *et al*., 2006; Wang *et al*., 2012).^1^ Decrease of either trehalose or mannitol leads to increased stress sensitivity of conidia (Fillinger *et al*., 2001; Ruijter *et al*., 2003; Sakamoto *et al*., 2008; Wang *et al*., 2012), indicating that both compounds may be important for stabilization of the biomolecules within these spores.

The protecting and stabilizing effect of compatible solutes is best studied for trehalose. This disaccharide is thought to have the most superior protective properties of any sugar (Crowe *et al*., 1984; Sola-Penna and Meyer-Fernandes, 1998; Kaushik and Bhat, 2003). By virtue of its unique α,α-1,1 glycosidic linkage, trehalose is a non-reducing sugar. Thus, trehalose is relatively unreactive, a prerequisite for successful stabilization. Trehalose provides protection for microbial cells, as well as enzymes, membranes and DNA *in vitro* (Crowe *et al*., 1984; Yoshinaga *et al*., 1997; Kandror *et al*., 2002; Jain and Roy, 2010) against a wide variety of stresses, including heat, freezing, desiccation, radiation and oxidative stress (Hottiger *et al*., 1989; Wiemken, 1990; Devirgilio *et al*., 1994; Yoshinaga *et al*., 1997; An *et al*., 2000; Benaroudj *et al*., 2001; Fillinger *et al*., 2001). Trehalose also protects cells against the chaotropicity-mediated stresses of substances such as hydrocarbons and solvents including toluene and ethanol (Bhaganna *et al*., 2010). The protective properties of trehalose are thought to be based on several principles including a high glass transition temperature (T_g_) (Sun and Davidson, 1998; Buitink and Leprince, 2004), the ability to replace water using its hydroxyl groups (water-replacement hypothesis) (Crowe *et al*., 1984; Crowe and Crowe, 1992) and its stabilizing effect on the water structure and intermolecular interactions in biomolecules as a result of preferential exclusion (Timasheff, 2002; Moelbert *et al*., 2004; Jain and Roy, 2009; Cray *et al*., 2013a).

A biological glass provides stability to a cell by significantly reducing molecular degrees of freedom (Crowe *et al*., 1998). Glass formation (vitrification) occurs during drying, or by rapidly cooling, and depends on the concentration of the solutes and the amount of water present (water acts as plasticizer) (Roos and Karel, 1990; Wolkers *et al*., 1998). The temperature also influences the melting of the glass (T_g_). Trehalose has a high glass transition temperature (T_g_ = 108°C) compared with other disaccharides (e.g. 67°C in case of sucrose), and it readily forms a glass at room temperature with a water content of 10% (Chen *et al*., 2000). Trehalose also has a larger binding capacity of water molecules than other disaccharides (Lerbret *et al*., 2005; Choi *et al*., 2006), despite having the same number of hydroxyl groups. This indicates that trehalose interacts more favourably with water molecules, which may also explain why it is such a good surrogate for water during desiccation. Trehalose is a macromolecule-structuring solute with a kosmotropic (structuring) activity of 10.6 kJ kg mole^−1^, which is almost twice that of compatible solutes as proline and mannitol (Cray *et al*., 2013a). This preferential exclusion stabilizes hydrophobic interactions and minimizes the surface area of proteins during the process of drying, thus preventing denaturation and loss of function (Elbein *et al*., 2003; Jain and Roy, 2010). Polyols and other oligosaccharides (consisting of two or more moieties) also protect against various stresses. For example, the polyol mannitol provides excellent protection against heat inactivation in solution (Ortbauer and Popp, 2008), but due to its limited solubility and tendency to crystallize, mannitol gives poor protection against both osmotic stress and (freeze) drying (Izutsu *et al*., 1993; Hallsworth and Magan, 1994; Al-Hussein and Gieseler, 2012).

Here, we describe the prevalence of novel trehalose-based oligosaccharides (TOS) in ascospores of *N. fischeri* as well as other fungal species which are abundantly present in the ascospores of some fungal species in the order Eurotiales. These TOS are characterized by their non-reducing nature and high glass-transition temperatures, which are proposed to protect the cells against drought and heat.

## Results

### Heat resistance of ascospores

**Figure 1:**
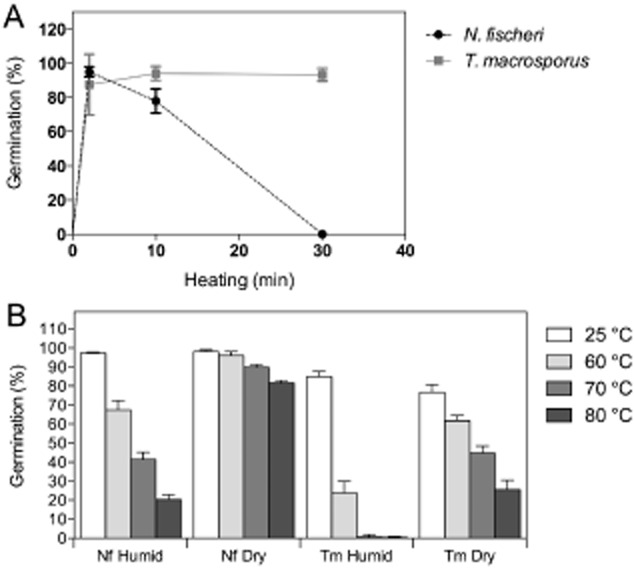
Germination (% of total) of *N*. *fischeri* and *T*. *macrosporus* ascospores after heating for 0–30 min at 85°C in ACES buffer (A) or after drying and storage for 1 week at 22–25°C at a RH of 45–85% (humid) or 0.5–2% (dry), a 1 h exposure at 25°C, 60°C, 70°C or 80°C in the absence of water, and a heat activation for 2 min at 85°C in ACES buffer (B).

Without any heat pre-treatment, *N. fischeri* ascospores have a low germination rate (0.33 ± 0.19%, Fig. 1A). Several seconds of heat exposure at 85°C is already sufficient to partially activate germination. Over 50% of the ascospores germinated after a heat treatment as short as 20 s at 85°C. Maximal activation (95.1 ± 2.9%) of *N. fischeri* ascospores was observed with a 2 min treatment at 85°C (Fig. 1A). No *N. fischeri* spores survived after a 30 min treatment at 85°C. Similar results were obtained during heat activation of *T. macrosporus* ascospores. Germination of *T. macrosporus* ascospores was not observed without heating (Fig. 1A). Partial activation occurred by a 20 s heat treatment (not shown), while maximal activation (94.1 ± 4.1%) was obtained after a heat treatment of 10 min. In contrast to *N. fischeri*, *T macrosporus* spores even survived a heat treatment of 30 min (germination 93.3 ± 3.8%).

In the next set of experiments, the effects of drying and heating *N. fischeri* and *T. macrosporus* ascospores were tested. The ambient-dry spores were vacuum dried for 1 h and kept for 7 days at 22–25°C with a RH of 45–85%, while the silica-dry spores were treated similarly but kept at a RH of 0.5–2%. After drying, the spores were incubated for 1 h at 60°C, 70°C or 80°C (dry heat), and their viability was measured microscopically after heat activation in an *N*-(2-acetamido)-2-aminoethanesulfonic acid (ACES) buffer for 2 min at 85°C. The silica-dry spores of *N. fischeri* and *T. macrosporus* were generally less sensitive to heat than the ambient-dry spores (Fig. 1B). Ascospore germination of *N. fischeri* was 97 ± 0.4% and 98 ± 0.9% in the case of ambient and silica-dried spores, respectively, kept at 25°C. These values were 85 ± 2.8% and 77 ± 4.0% for ascospores of *T. macrosporus* respectively. Heat treatment for 1 h at a higher temperature resulted in decreased germination. *Neosartorya fischeri* ascospores stored at ambient RH showed 68 ± 4.9%, 42 ± 3.7% and 20 ± 2.5% germination when incubated at 60°C, 70°C and 80°C respectively. The silica-dry *N. fischeri* ascospores showed higher survival rates (96 ± 2.1%, 90 ± 1.5% and 82 ± 1.3% respectively). Germination of *T. macrosporus* ascospores was more significantly affected by the heat treatments. Exposure at 60°C, 70°C and 80°C resulted in germination of 24 ± 6.2%, 0.6 ± 1.1% and 0.3 ± 0.6% for the ambient-dry spores and 62 ± 2.6%, 45 ± 3.8 and 26 ± 4.9% for the silica-dry ascospores respectively.

### Microviscosity of ascospores

**Figure 2:**
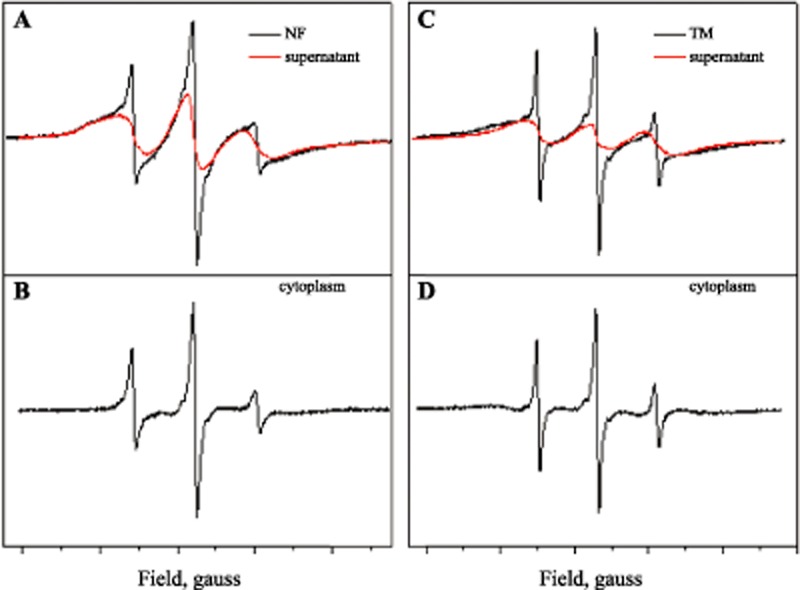
The intensity of the ESR spectra obtained from ascospores of *N*. *fischeri* (A, B) and *T*. *macrosporus* (C, D) that were labelled with the spin probe TEMPONE. The spectra are composed of a signal originating from the cell wall and the medium (A, C) and an intracellular signal. The latter is calculated by subtracting the signal of the cell wall and the medium from the total signal (B, D). Supernatant is the extracellular solution in which the spores are suspended and composed of demi water, TEMPONE and FC (ferricyanide).

Electron spin resonance (ESR) spectra of ascospores containing the spin probe 4-oxo-2,2,6,6-tetramethylpiperidine-N-oxy (TEMPONE) were used for calculation of the (micro)viscosity of the cytoplasm. These spectra are a superposition of broad and narrow-line spectra. The narrow-line spectrum originates from TEMPONE that is present inside the cell. The broad component is a signal from TEMPONE/ferricyanide (FC) that is located extracellularly (residing in the cell wall and the medium). This component has to be subtracted from the recorded spectrum to obtain the narrow line spectrum, from which the microviscosity can be calculated (Fig. 2; Table 1). The calculated microviscosities before heating and cooling were 15.8 and 10.5 cP for *N. fischeri* and *T. macrosporus* ascospores respectively (Table 1). These values were 14.2 and 9.8 cP after heating and cooling respectively. The subsequent ESR spectra remained intact and still contained narrow lines. However, the signal was less intense, which indicates a reduction of the amount of paramagnetic spin-probe molecules.

**Table 1:** Effective cytoplasmic viscosity calculated from the rotational correlation time of intracellular TEMPONE of *N*. *fischeri* (Nf) and *T*. *macrosporus* (Tm) ascospores

	Rotation correlation time (s)		Viscosity (cP)	
	Before heating	After heating	Before heating	After heating
Nf	4.3 10^−10^	3.9 10^−10^	15.8	14.2
Tm	2.9 10^−10^	2.7 10^−10^	10.5	9.8
water	0.24 10^−10^	n.a.	0.89	n.a.

### Identification of oligosaccharides in ascospores

**Figure 3:**
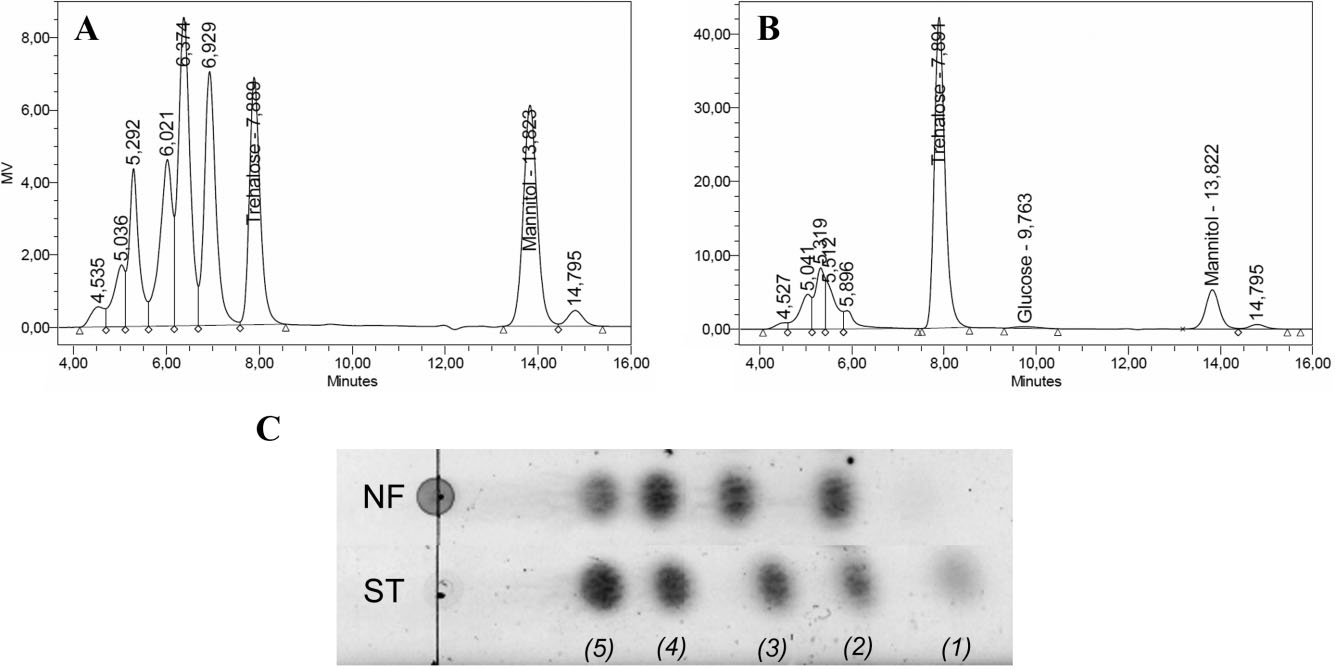
HPLC profiles of cell-free extracts of *N*. *fischeri* (A) and *T*. *macrosporus* (B) ascospores that had been isolated from 40-day-cultures. Besides trehalose and mannitol, the *N*. *fischeri* extract has peaks with shorter retention times than trehalose (C) TLC of *N*. *fischeri* ascospore extract (NF) and a sugar standard (ST) consisting of glucose (1), trehalose (2), raffinose (3), stachyose (4) and verbascose (5). *Neosartorya fischeri* spores contain oligosaccharides of different size: a disaccharide, trisaccharide, tetrasaccharide and pentasaccharide.

Cell-free extracts of ascospores from 40-day-old cultures of three independent isolates of *N. fischeri* (Fig. 3A) and *T. macrosporus* (Fig. 3B) were analysed by high-performance liquid chromatography (HPLC) to identify compatible solutes. The elution patterns of the isolates of *T. macrosporus* were dominated by a peak with the same retention time as trehalose (RT 7.9 min). In addition, a small peak at the position of mannitol (RT 13.8 min) was observed. The cell-free extract of *N. fischeri* also showed trehalose and mannitol peaks that were significantly lower and higher, respectively, than that of *T. macrosporus*. The HPLC spectrum of *N. fischeri* was characterized by three additional peaks with a RT of 6.0, 6.4 and 6.9 min (Fig. 3A).

The three additional peaks were identified as tri- tetra- and pentasaccharides respectively by means of nuclear magnetic resonance (NMR) spectroscopy (Wyatt, 2014). Based on this study, the primary structure of the saccharides has been found to contain an α,α-1,1 trehalose core, with one, two or three α-1,6 linked glucose extensions. These molecules, collectively dubbed as TOS, are called isobemisiose (trisaccharide), neosartose (tetrasaccharide) and fischerose (pentasaccharide). With thin layer chromatography (TLC), the trehalose and the other oligosaccharides had similar retention times as the plant disaccharides sucrose, the trisaccharide raffinose, the tetrasaccharide verbascose and the pentasaccharide stachyose (Fig. 3C).

### Quantification of soluble sugars and polyols in ascospore extracts

**Figure 4:**
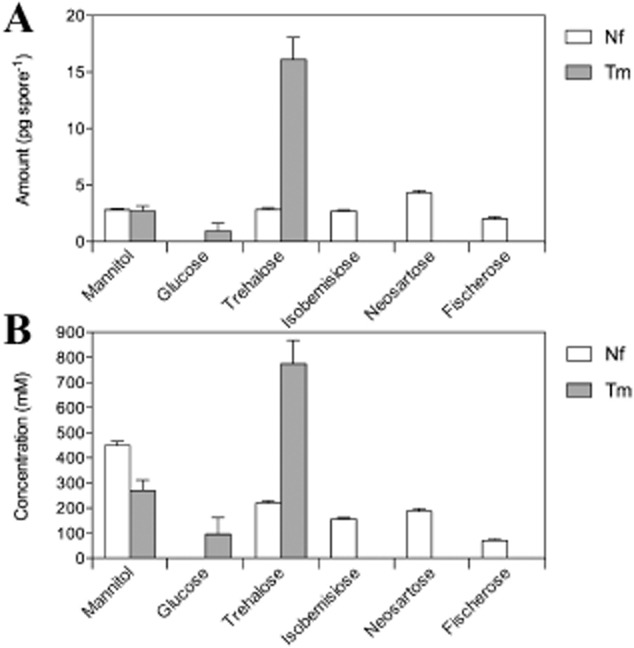
Compatible solutes in *N*. *fischeri* (non-shaded bars) and *T*. *macrosporus* (grey-shaded bars) ascospores expressed as pg spore^−1^ (A) and mM (B).

The amount of compatible solutes in ascospores (Fig. 4A) was calculated from HPLC analyses using calibration curves as described in *Experimental procedures*. For the quantification of the newly discovered oligosaccharides, we used synthesized oligosaccharides for calibration. Ascospores of a 40-day-old culture of *N. fischeri* accumulated 2.8 ± 0.2 pg spore^−1^ mannitol, 2.8 ± 0.2 pg spore^−1^ trehalose, 2.7 ± 0.2 pg spore^−1^ of the trisaccharide, 4.3 ± 0.4 pg spore^−1^ of the tetrasaccharide and 2.0 ± 0.3 pg spore^−1^ of the pentasaccharide. Ascospores of the fungus *T. macrosporus* accumulated mainly trehalose (16.1 ± 3.4 pg spore^−1^) with 2.7 ± 0.7 pg spore^−1^ mannitol and 1.0 ± 1.2 pg spore^−1^ glucose. To calculate the concentration of the compatible solutes in the ascospores, the dimensions of the ascospores were determined. To this end, dimensions of 101 and 45 ascospores of *N. fischeri* and *T. macrosporus* were measured by light microscopy respectively. The cell-wall thickness of ascospores of 40-day-old cultures of *N. fischeri* was measured using staining with carboxyfluorescein, while autofluorescence of the cell wall was used in the case of *T. macrosporus* (Fig. S1). *Neosartorya fischeri* ascospores have the shape of a sphere (V = 4/3 πr^3^). The cell has a diameter of 4.0 ± 0.2 µm (r = 2 µm), excluding a cell wall that is approximately 0.3 µm thick. The shape of the *T. macrosporus* ascospores is a prolate spheroid (V = 4/3 πab^2^, with a = long and b = short dimension) (Dijksterhuis and Samson, 2002). The cellular volume enclosed by the cell wall has a long diameter of 5.2 ± 0.3 µm (a = 2.6 µm) and a short diameter of 4.5 ± 0.2 µm (b = 2.3 µm). The cell wall was approximately 0.4 µm thick (ornamentation not included). These data result in a cytoplasmic volume of 34 ± 4 fl and 55 ± 8 fl for *N. fischeri* and *T. macrosporus* ascospores respectively. Taking these values into account, *N. fischeri* ascospores contain 449 ± 28 mM mannitol (Fig. 4B). The concentration of the sugars was less for trehalose, and the tri-, tetra- and pentasaccharide, being 220 ± 17 mM, 156 ± 11 mM, 187 ± 18 mM and 70 ± 11 mM respectively. The ascospores of *T. macrosporus* contained 269 ± 27 mM mannitol, 774 ± 163 mM trehalose and 96 ± 115 mM glucose. *Talaromyces macrosporus* samples showed considerable variation in the accumulation of compatible solutes, especially in the amount of glucose. This could be due to variations in trehalose degradation by an active trehalase (Dijksterhuis and Samson, 2002; Dijksterhuis *et al*., 2002) after breaking of ascospores. Alternatively, glucose could function as a compatible solute by itself (Jennings and Burke, 1990).

### Glass transition temperature and density of sugar/polyol solutions

**Figure 5:**
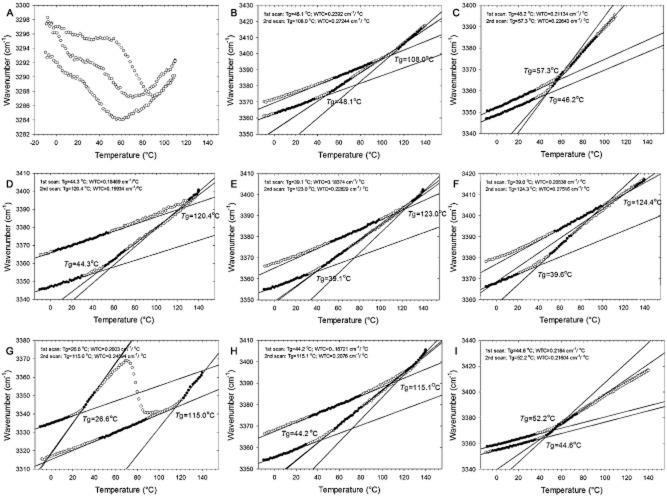
Linear regression of the wavenumber of the OH stretching band as function of the temperature of the samples consisting of mannitol (A), trehalose (B), sucrose (C), isobemisiose (D), neosartose (E), fischerose (F), trehalose/mannitol (G), mannitol/trehalose/isobemisiose/neosartose/fischerose (H) and mannitol/sucrose/raffinose/stachyose/verbascose (I). No regression line could be determined in the mannitol sample due to crystallization (A). Mannitol crystallization was also observed in the trehalose/mannitol mixture (G). The intersection of the regression lines represents the glass transition temperature with T_g1_ and T_g2_ determined from the 1st and 2nd scan respectively. The steepness of the regression line corresponds with the WTC value (cm^−1^ °C^−1^).

Samples composed of trehalose, isobemisiose, neosartose, fischerose, sucrose, stachyose, verbascose, mannitol and mixtures thereof were analysed by Fourier transform infrared (FTIR) spectrometry. Spectra were recorded from −10°C to 140°C, back from 140°C to −10°C and again from −10°C to 140°C. The glass transition temperature (T_g_) and the wave number-temperature coefficient (WTC) were deduced from the FTIR spectra (Fig. 5). WTC is defined as the rate of change of the vibrational energy with temperature (cm^−1^ °C^−1^). Notably, the T_g_ of the first series recorded from −10°C to 140°C, T_g1_, differed significantly from the second recording from −10°C to 140°C (note that this is the third series). The latter values (T_g2_), when known (e.g. trehalose, raffinose), are in line with published values (e.g. Wolkers *et al*., 1998). For both fungal and plant sugars, T_g2_ increased with the amount of sugar groups. In the case of the fungal and plant sugars, the following order was observed: trehalose < isobemiose < neosartose < fischerose and sucrose < raffinose < stachyose < verbascose (Table 2). The T_g2_ of sucrose had the lowest value (56.6°C), while fischerose had the highest T_g2_ value (124.4°C). Notably, the T_g2_ of the plant sugars were lower compared with their fungal counterparts. For instance, T_g2_ of sucrose is 42°C lower than that of trehalose, and the T_g2_ of raffinose is 13°C lower than that of isobemisiose (Table 2). Moreover, mixtures of trehalose and mannitol with the fungal tri-, tetra- and pentasaccharides had a much higher T_g2_ compared with these mixtures with the plant counterparts.

**Table 2:** T_g_ (glass-transition temperature) and WTC (wave number-temperature coefficient) of mannitol (Man), trehalose (Tre), isobemisiose (Iso), neosartose (Neo), fischerose (Fis), sucrose (Suc), raffinose (Raf), stachyose (Sta), verbascose (Ver) and mixtures thereof as determined by FTIR spectrometry

	T_g1_	T_g2_	WTC_1_	WTC_2_
Mannitol (Man)^a^	n.d.	n.d.	n.d.	n.d.
Trehalose (Tre)	48.1	108	0.239	0.272
Isobemisiose (Iso)	44.3	120.4	0.185	0.200
Neosartose (Neo)	39.1	123	0.184	0.228
Fischerose (Fis)	38.8	125.4	0.195	0.256
Tre + Man (1:1)	26.6	115	0.200	0.246
Iso + Neo + Fis (1:1:1)	43.1	123.4	0.172	0.234
Tre + Iso + Neo + Fis (1:1:1:1)	40.6	122.8	0.175	0.223
Tre + Iso + Neo + Fis + Man (1:1:1:1:1)	44.2	115.1	0.187	0.207
Iso + Neo + Fis + Man (1:1:1:1)	43.4	52.7	0.185	0.208
Sucrose (Suc)	43.8	65.6	0.215	0.200
Raffinose (Raf)	40.1	107.8	0.213	0.264
Stachyose (Sta)	39.5	119.2	0.207	0.225
Verbascose (Ver)	33.8	123.8	0.213	0.273
Suc + Man (1:1)	21.6	63	0.200	0.179
Raf + Sta + Ver (1:1:1)	45.8	123.2	0.217	0.262
Suc + Raf + Sta + Ver (1:1:1:1)	41.2	98.1	0.213	0.252
Suc + Raf + Sta + Ver + Man (1:1:1:1:1)	44.6	52.2	0.218	0.216

a Due to crystallization of mannitol no values could be obtained.

The WTC value represents the strength of hydrogen bonding in a glass. High WTC values indicate weaker hydrogen bonding, and a glass that has a higher degree of freedom for rearrangement (Wolkers *et al*., 2004). Thus, higher WTC values indicate a less dense glass. Trehalose, raffinose, verbascose and fischerose showed the highest WTC_2_ values. Glasses of other pure solutes or mixtures had lower values with the lowest value for the sucrose/mannitol mixture (Table 2). This indicates that the glass of the sucrose/mannitol mixture has the highest density.

T_g1_ and T_g2_ values were highly different (Table 2). The T_g1_ values ranged between 21.6°C and 48.1°C for trehalose and the sucrose/mannitol glass respectively. T_g1_ decreased with increasing degree of polymerization (DP) for both fungal and plant sugars: trehalose > isobemiose > neosartose > fischerose and sucrose > raffinose > stachyose > verbascose (Table 2). The mixtures had a relatively high T_g1_ value. These data suggest that glass formation has a large influence on T_g_, and henceforth the protective properties of a glass. The same holds for the WTC parameters. The WTC_1_ values are all lower than the WTC_2_ values. The highest values existed for TOS and sucrose-based oligosaccharides (SOS) (0.207–0.239 cm^−1^ °C^−1^), and the lowest values for TOS and TOS mixtures (0.172–0.195 cm^−1^ °C^−1^). The lower WTC values of the TOS indicate tighter packed hydrogen bonds, and a more densely packed glass structure.

**Figure 6:**
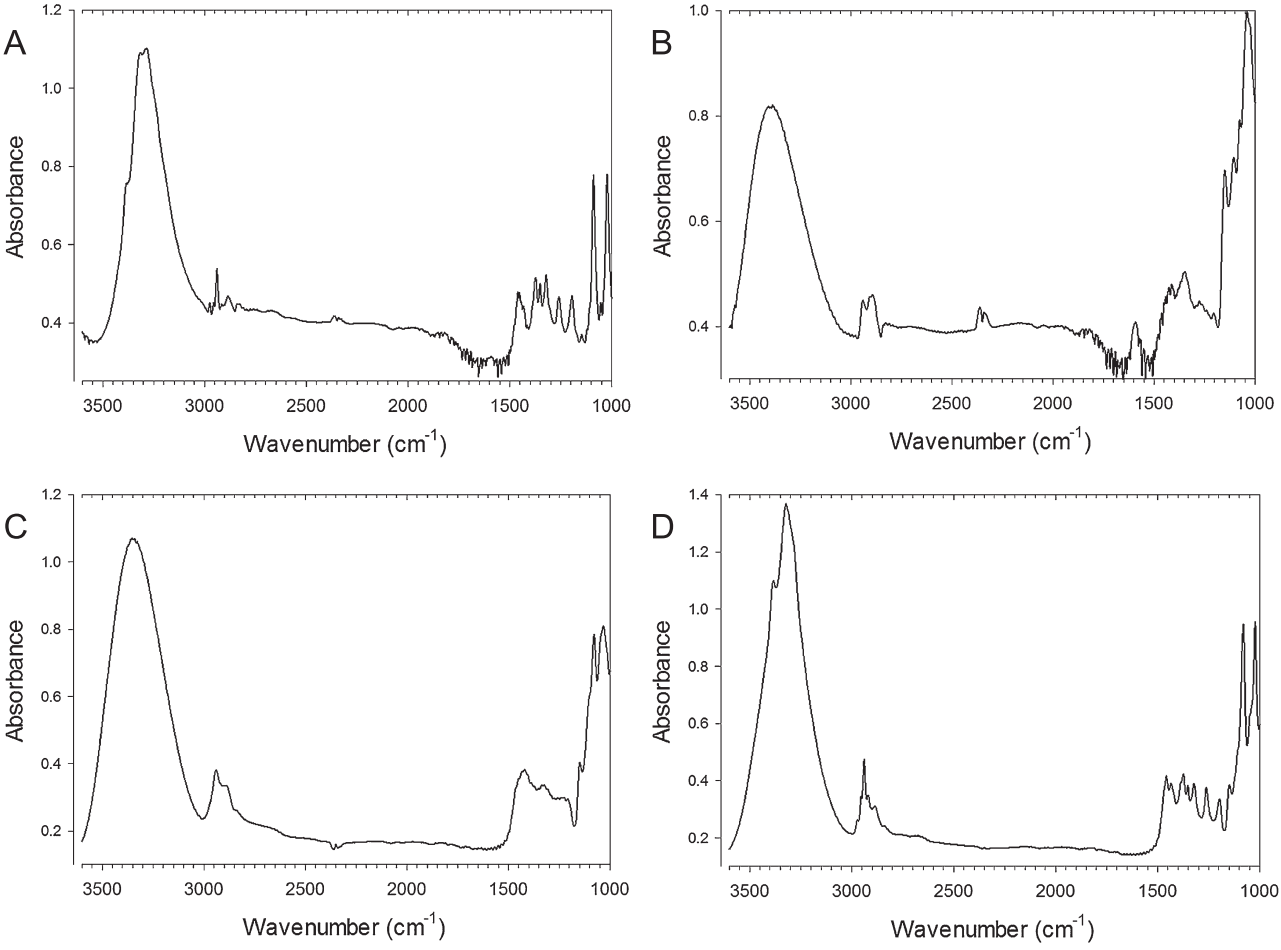
Absorbance spectrum of mannitol (A), fischerose (B) and trehalose + mannitol (C, D) as measured by FTIR. The spectra of A, B and D are from the 2nd scan (after heating and re-cooling), while C is of the 1st scan. The change of peak shape of the trehalose + mannitol sample (C, D) is due to crystallization. The mannitol sample (A), but not fischerose (B), also shows crystallization.

Regression lines could not be obtained for T_g_ or WTC in the case of mannitol, trehalose/mannitol and sucrose/mannitol samples (Fig. 6). The sharp peak (around 3250 cm^−1^) on the FTIR absorbance spectra of these samples indicates that mannitol crystallization had occurred when the 2nd scan was taken (Fig. 6A and D). The peaks of fischerose (Fig. 6B) or the 1st scan of the mannitol/trehalose (Fig. 6C) sample are less sharp and indicate an amorphous state. The presence of mannitol results in a strong decrease of the T_g2_ values of several sugar mixtures, yet this effect was low in the case of the sugar mixtures observed in *N. fischeri* ascospores. This suggests that the tendency of mannitol to crystallize is suppressed in mixtures of TOS, but not (or less) in mixtures of SOS such as the raffinose family oligosaccharides (RFOs).

### Occurrence of TOS in species of the order Eurotiales

**Figure 7:**
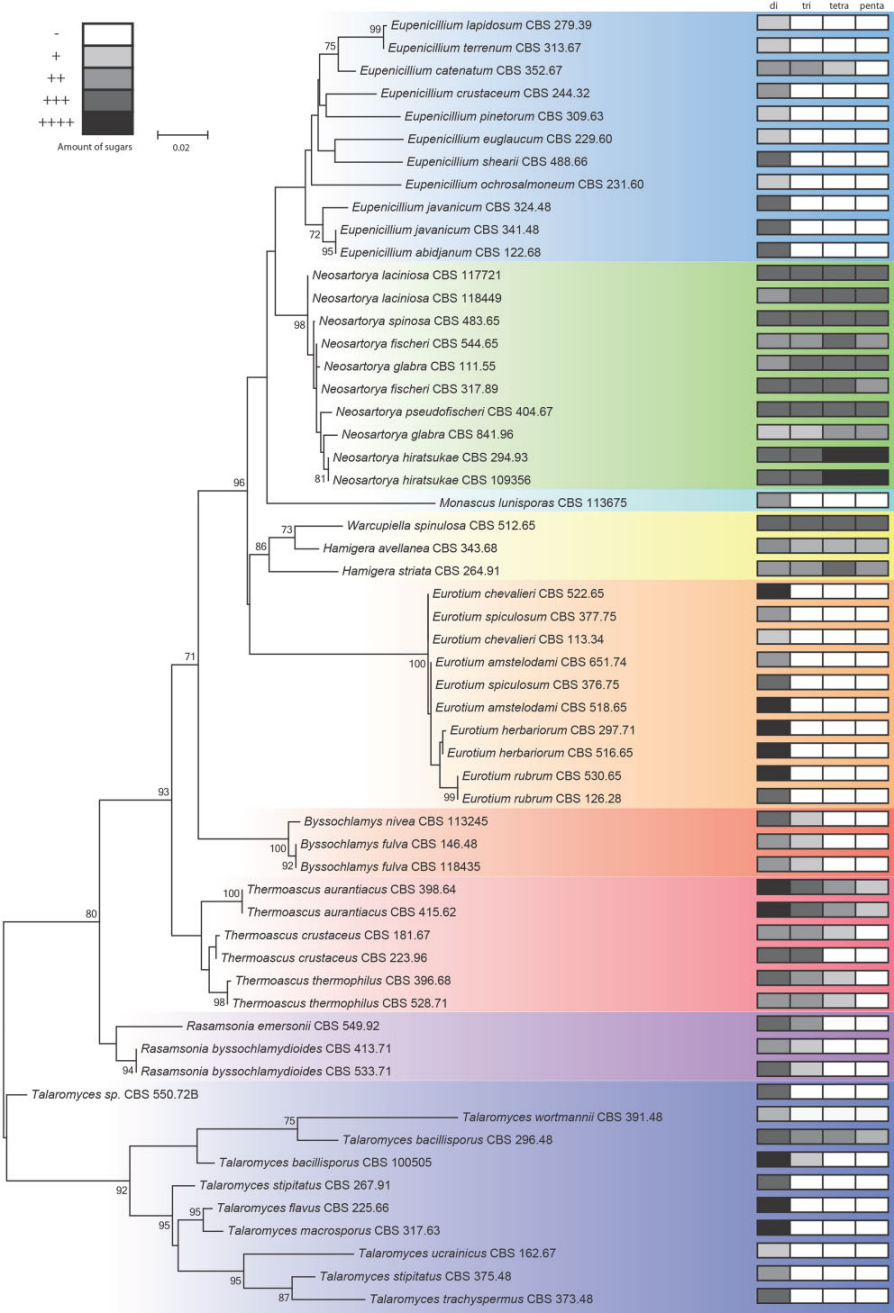
Phylogenetic tree of the family *Trichocomaceae* indicating the presence or absence of trehalose and TOS in the ascospores. The presence of oligosaccharides is indicated in the boxes (labelled with di-, tri-, tetra- and penta- for corresponding oligosaccharides) at the right side of the Figure. (Oligo)saccharides were measured by TLC and the grey scale indicates the intensity of staining of the TLC band.

In order to evaluate if TOS are occurring more widespread among fungi, a multilocus phylogenetic tree was constructed with DNA sequences from the ribosomal internal translated spacer (ITS) and ribosomal large subunit (LSU) obtained from various Eurotiales including the genera *Byssochlamys*, *Eupenicillium* (*Penicillium*), *Eurotium*, *Hamigera/Warcupiella*, *Monascus*, *Neosartorya* (*Aspergillus*), *Rasamsonia*, *Talaromyces* and *Thermoascus* (Fig. 7). This selection includes well-known producers of extreme heat-resistant ascospores such as *Byssochlamys nivea*, *Byssochlamys fulva*, *T. macrosporus*, *Talaromyces flavus* and *N. fischeri*. The presence of oligosaccharides in the ascospore extracts was analysed by TLC. Note that polyols such as mannitol cannot be detected with this method. All six species (10 strains) tested of the genus *Neosartorya* showed an oligosaccharide TLC pattern identical to that of *N. fischeri*. HPLC analysis of the ascospore extracts confirmed the presence of isobemisiose, neosartose and fischerose (Table 3). According to TLC, three species of the *Hamigera/Warcupiella* clade also contained four oligosaccharides, with the same retention time as trehalose, isobemisiose, neosartose and fischerose (Fig. 7). Two *Byssochlamys* and two *Rasamsonia* species contained a disaccharide and trisaccharide, and ascospores of three *Thermoascus* species accumulated oligosaccharides larger than a disaccharide. *Thermoascus aurantiacus* accumulated a di-, tri-, tetra- and pentasaccharide, while *Thermoascus thermophilis* strains and *Thermoascus crustaceus* accumulated a di- and trisaccharide or a di-, tri- and tetrasaccharide dependent on the strain used. In contrast, the majority of fungal species belonging to the genera *Eurotium*, *Eupenicillium*, *Monascus* and *Talaromyces* form ascospores that showed a band with the same retention time as trehalose. *Eupenicillium catenatum* and *Talaromyces bacillisporus* accumulated a di- and trisaccharide. Another isolate from *T. bacillisporus* (CBS 296.48) accumulated four oligosaccharides, which suggest the presence of a similar quartet of compounds observed within the genus *Neosartorya*. Sequencing revealed that the two *T. bacillisporus* strains were genetically different, which indicates different *Talaromyces* species. These data show that these oligosaccharides are a hallmark of the genus *Neosartorya*, but also occur in other groups within the order Eurotiales and the family Trichocomaceae. Interestingly, 13 out of 19 species that contained oligosaccharides inside ascospores apart from trehalose have maximal growth temperatures at or above 45°C (Table 3).

**Table 3:** Strains used to study occurrence of trehalose (Tre), isobemisiose (Iso), neosartose (Neo) and fischerose (Fis) in ascospore extracts

Species	Strain	Culture conditions		Maximal growth temp. (T_max_)	Proportion of sugars (% of total)			
	CBS #	Media	Temp. (°C)	°C	Tre	Iso	Neo	Fis
*Byssochlamys fulva*	132.33	OA	30	45^a^	n.d.	n.d.	n.d.	n.d.
*Byssochlamys fulva*	11845	OA	30	45^a^	n.d.	n.d.	n.d.	n.d.
*Byssochlamys nivea*	100.11	OA	30	40^a^	n.d.	n.d.	n.d.	n.d.
*Eupenicillium abidjanum*	122.68	OA	25	> 37^b^	100	0	0	0
*Eupenicillium catenatum*	352.67	OA	25	> 37^b^	56	23	13	7
*Eupenicillium crustaceum*	244.32	OA	25	< 37^b^	100	0	0	0
*Eupenicillium euglaucum*	229.6	OA	25		100	0	0	0
*Eupenicillium javanicum*	341.48	OA	25	> 37^b^	100	0	0	0
*Eupenicillium javanicum*	324.48	OA	25	> 37^b^	100	0	0	0
*Eupenicillium lapidosum*	279.39	OA	25		100	0	0	0
*Eupenicillium ochrosalmoneum*	231.60	OA	25	> 37^b^	100	0	0	0
*Eupenicillium pinetorum*	309.63	OA	25	< 37^b^	100	0	0	0
*Eupenicillium shearii*	488.66	OA	25	37^b^	100	0	0	0
*Eupenicillium terrenum*	313.67	OA	25	37^b^	100	0	0	0
*Eurotium amstelodami*	518.65	MEA40S	25	43–46^c^	100	0	0	0
*Eurotium amstelodami*	651.74	MEA40S	25	43–46^c^	100	0	0	0
*Eurotium chevalieri*	113.34	MEA40S	25	37–49^c^	100	0	0	0
*Eurotium chevalieri*	522.65	MEA40S	25	37–49^c^	100	0	0	0
*Eurotium herbariorum*	516.65	MEA40S	25	37–40^c^	100	0	0	0
*Eurotium herbariorum*	297.71	MEA40S	25	37–40^c^	100	0	0	0
*Eurotium rubrum*	530.65	MEA40S	25		100	0	0	0
*Eurotium rubrum*	126.28	MEA40S	25		100	0	0	0
*Eurotium spiculosum*	377.75	MEA40S	25		100	0	0	0
*Eurotium spiculosum*	376.75	MEA40S	25		100	0	0	0
*Hamigera avellanea*	343.68	OA	30		0	33	33	33
*Hamigera striata*	264.91	OA	30		n.d.	n.d.	n.d.	n.d.
*Monascus lunisporas*	113675	OA	25		n.d.	n.d.	n.d.	n.d.
*Warcupiella spinulosa*	512.65	OA	25		n.d.	n.d.	n.d.	n.d.
*Neosartorya fischeri*	544.65	OA	30	51–52^c^	24	20	34	22
*Neosartorya fischeri*	317.89	OA	30	51–52^c^	28	26	32	14
*Neosartorya glabra*	111.55	OA	30		18	27	34	21
*Neosartorya glabra*	841.96	OA	30		17	17	33	33
*Neosartorya hiratsukae*	294.93	OA	30	43^d^	15	16	34	36
*Neosartorya hiratsukae*	109356	OA	30	43^d^	20	15	34	31
*Neosartorya laciniosa*	117721	OA	30	> 45 < 50^e^	24	27	32	18
*Neosartorya laciniosa*	118449	OA	30	> 45 < 50^e^	17	27	33	24
*Neosartorya pseudofischeri*	404.67	OA	30		34	24	27	15
*Neosartorya spinosa*	483.65	OA	30		25	25	30	20
*Rasamsonia byssochlamydioides*	413.71	OA	40		n.d.	n.d.	n.d.	n.d.
*Rasamsonia byssochlamydioides*	533.71	OA	40		n.d.	n.d.	n.d.	n.d.
*Rasamsonia emersonii*	549.92	OA	40	55^f^	79	21	0	0
*Talaromyces bacillisporus*	296.48	OA	36	45^f^	47	30	15	8
*Talaromyces bacillisporus*	100505	OA	36	45^f^	100	0	0	0
*Talaromyces flavus*	225.66	OA	30	40^f^	100	0	0	0
*Talaromyces macrosporus*	317.63	OA	30	40^f^	100	0	0	0
*Talaromyces sp*.	550.72B	OA	30	< 40^f^	100	0	0	0
*Talaromyces stipitatus*	375.48	OA	30	< 40^f^	100	0	0	0
*Talaromyces stipitatus*	267.91	OA	30	< 40^f^	100	0	0	0
*Talaromyces trachyspermus*	373.48	OA	30	40^f^	100	0	0	0
*Talaromyces ucrainicus*	162.67	OA	30	< 40^f^	100	0	0	0
*Talaromyces wortmannii*	391.48	OA	30	< 40^f^	100	0	0	0
*Thermoascus aurantiacus*	398.64	OA	40	55–62^c^	75	18	6	1
*Thermoascus aurantiacus*	415.62	OA	40	55–62^c^	69	17	7	8
*Thermoascus crustaceus*	181.67	OA	40	55^g^	n.d.	n.d.	n.d.	n.d
*Thermoascus crustaceus*	223.96	OA	40	55^g^	63	37	0	0
*Thermoascus thermophilus*	528.71	OA	40		70	30	0	0
*Thermoascus thermophilus*	396.68	OA	40		72	28	0	0

References T_max_:

a Houbraken (unpublished);

b Pitt (1979);

c Domsch and colleagues (1980);

d Koutroutsos and colleagues (2010);

e Malejczyk and colleagues (2013);

f Stolk and Samson (1972);

g Morgenstern and colleagues (2012).

The proportions of sugars are based on their occurrence in HPLC profiles in which the pure (oligo)saccharides were used for calibration. A number of the strains are not determined.

## Discussion

Here we report the prevalence of TOS as abundant compatible solutes in fungi, namely in the ascospores of *N. fischeri* and related species. Our data suggest that these oligosaccharides are widespread in the order Eurotiales. The molecules have a trehalose core and one to three glucose moieties are linked to one side of the molecule via α-1,6 glycosidic linkages (Wyatt, 2014). While the occurrence of isobemisiose has previously been reported in the whitefly *Bemisia argentifolii* (Hendrix and Salvucci, 2001), neosartose and fischerose are reported in this study for the first time as naturally occurring oligosaccharides.

TOS distinct from isobemisiose, neosartose and fischerose that do not have α-1,6 glycosidic linkages have been described earlier in several organisms including insects (*B. argentifolii*; Hendrix and Salvucci, 2001); bacteria (*Mycobacterium smegmatis*, Besra *et al*., 1993; Ohta *et al*., 2002; Tropis *et al*., 2005; *Sinorhizobium meliloti*, Hisamatsu *et al*., 1985; Breedveld and Miller, 1994; Brique *et al*., 2010); yeast (*Saccharomyces cerevisiae*, Iwahara *et al*., 1993). *Sinorhizobium meliloti* accumulates a number of TOS when grown in hyperosmolarity (Brique *et al*., 2010). Brique and co-workers suggest that these TOS act as precursors of trehalose. It has been suggested that chaotropic environments may potentially harbour chaotrope-tolerant, or even chaophilic, species of microbe (Williams and Hallsworth, 2009; Hallsworth *et al*., 2007; Leong *et al*., 2014; Lievens *et al*., 2014; Oren and Hallsworth, 2014). Whereas there is currently no definitive evidence for the existence of chaophilic microbes, the chaotrope-tolerance mechanisms that have been proposed in such studies (see also Hallsworth, 1998; Cray *et al*., 2013b; Yakimov *et al*., 2014) would most likely include accumulation of highly kosmotropic compatible solutes such as TOS.

In plants, oligosaccharides are also accumulated, including the SOS, fructans and the RFOs (Valluru and van den Ende, 2008). The latter molecules, like TOS, contain a disaccharide core, but in this case sucrose (i.e. glucose linked to fructose via an α-1,2-β glycosidic linkage). Fructans are fructose polymers with a sucrose molecule linked at the reducing end. The RFOs consist of sucrose with one, two or three galactose moieties linked α-1,6 to the glucose moiety of sucrose, and called raffinose, stachyose and verbascose respectively. There is a striking similarity to the fungal TOS homologues where one, two or three glucose moieties are connected with an α-1,6 linkage. The functionality of the fungal TOS is not studied in great detail; however, fructans and RFOs are believed to act as membrane protectors under stress (Hincha *et al*., 2002,2003) via direct hydrogen binding (Milhaud, 2004; Beck *et al*., 2007). A flexible bond between the saccharide moieties, such as the α-1,6 glycosidic linkage, could be essential for insertion of the sugars between the lipids (Valluru and van den Ende, 2008). Other authors claim that fructans and RFOs protect against oxidative stress and lipid oxidation (Cacela and Hincha, 2006; Agati *et al*., 2007; Nishizawa *et al*., 2008; Van den Ende and Valluru, 2009). The concentration of raffinose, stachyose and verbascose during maturation of plant seeds increases (Kuo *et al*., 1988; Blackman *et al*., 1992; Bernallugo and Leopold, 1995). Seed maturation, in turn, is correlated with increased stress tolerance, longevity and glass formation (Brenac *et al*., 1997).

Thus, like SOS, it can be assumed that TOS also protect against abiotic stress. What is the advantage of accumulation of isobemisiose, neosartose, fischerose, trehalose and mannitol in a mixture compared to trehalose alone? Indeed, ascospores of *T. macrosporus* (trehalose accumulation) survived 1 h at 85°C, while *N. fischeri* (TOS accumulation) could not. Strikingly, ascospores of *N. fischeri* survived desiccation and dry heat better than *T. macrosporus*. This suggests that TOS may function in the protection of spores against drought and subsequent heat. Upon drying, the interior of the ascospore forms a biological glass, and the properties of this glass are expected to function in spore survival during prolonged periods of high ambient (between 30°C and 50°C) temperatures. The cytoplasm of ascospores is a matrix of proteins, membranes, nucleic acids, organic and inorganic acids, and sugars with different polymerizations. The stabilizing glass formed in ascospores during drying has properties that differ from glasses composed of mixtures of oligosaccharides *in vitro*. Glass properties in plants corresponds more to a glass composed of a sugar/protein mixture than solely a mixture of oligosaccharides (Buitink and Leprince, 2008). Other molecules (e.g. inorganic molecules, organic acids and amino acids) also influence the properties of the glass, as was shown for citrate (Kets *et al*., 2004). The molecules that contribute to glass formation and its properties collectively determine their protective capacity. Thus, the context of the TOS inside the ascospores impacts their properties and thereby their function in the cell. In addition, the TOS will tend to have more protective activity (including kosmotropic activity) when compared on a molar basis as is observed for polyethylene glycol and other polymers (Cray *et al*., 2013a) without a drastic reduction of the intracellular water activity that would otherwise induce a self-imposed osmotic stress. This is relevant in case of *N. fischeri* where accumulation of solutes during maturation of the ascospores occurs without obvious external osmotic stress (Wyatt *et al*., 2014). Physiologically active cells of microbial species that are able to inhabit types of hypersaline, high-sugar or alcohol-containing experience conditions that are highly chaotropic.^2^ Whereas there is not yet any definitive evidence for the existence of chaophilic microbes (Hallsworth *et al*., 2007; Williams and Hallsworth, 2009; Leong *et al*., 2014), the chaotrope-tolerance mechanisms that have been proposed in these studies (see Hallsworth *et al*., 2007; Yakimov *et al*., 2014) would most likely include accumulation of highly kosmotropic compatible solutes such as TOS.

FTIR experiments showed that glasses prepared from pure oligosaccharides or mixtures of TOS, trehalose and/or mannitol behave differently after heating during the first scan. T_g1_ values are invariably lower than the literature (T_g2_) values. Heating in the first scan could remove any residual water molecules and/or rearrange the molecules in the glass, leading to the so-called ‘matured’ glass, which has a higher melting temperature. As expected, T_g2_ correlates with the DP (Table 3). To our surprise, T_g1_ seems to be negatively correlated with the DP. One possible explanation for this could be that glass ‘maturation’ occurs faster in glasses composed of lower DP molecules. The T_g1_ of the TOS was higher than the value corresponding to the plant homologues. Mannitol did not crystallize in a TOS/trehalose mixture, but did so in a 1:1 mannitol/trehalose and mannitol/sucrose mixture. In these complex mixtures as those occurring in ascospores of *N. fischeri*, the proportion of mannitol is lower than in 1:1 mixtures and we hypothesize that this prevents crystallization of the compound. In addition, the T_g_ of mannitol/TOS mixtures was much higher than the mannitol/RFO mixtures. Mannitol serves as an important protectant in water-containing environments. Furthermore, with its small size, it costs less carbon to synthesize mannitol compared with the saccharide molecules. Trehalose seems to be most effective in a dry state or in the phase between hydrated and dehydrated (low humidity). The combination of these compatible solutes might provide protection at strongly fluctuating water availabilities.

It has been suggested that chaotropic environments may potentially harbour chaotrope-tolerant, or even chaophilic, species of microbe (Williams and Hallsworth, 2009; Hallsworth *et al*., 2007; Leong *et al*., 2014; Lievens *et al*., 2014; Oren and Hallsworth, 2014). Whereas there is currently no definitive evidence for the existence of chaophilic microbes, the chaotrope-tolerance mechanisms that have been proposed in such studies (see also Hallsworth, 1998; Cray *et al*., 2013b; Yakimov *et al*., 2014) would most likely include accumulation of highly kosmotropic compatible solutes such as TOS. The presence of TOS does correlate in most cases with a thermophilic or thermotolerant nature of fungi. A fungus is regarded as thermophilic when it can grow at or above 50°C, but not below 20°C. Thermotolerant species have a maximum growth temperature of 45–50°C, and a minimum growth temperature below 20°C (Cooney and Emerson, 1964). For instance, *Neosartorya*, *Thermoascus* and *Rasamsonia* are well-known thermotolerant/thermophilic fungi (Mouchacca, 1997,2007; Houbraken *et al*., 2012a,2012b). *Byssochlamys* species are also known to be moderately thermotolerant and are able to grow at temperatures above 40°C. *Hamigera* species can be found in hot climates and several of these species are known to be able to grow above 40°C. *Talaromyces bacillisporus*, which also seem to accumulate TOS, is also moderately thermotolerant (Stolk and Samson, 1972). This indicates the fungal species that form complex mixtures of compatible solutes in their survival structures tend to occur in areas with higher temperatures. In the occurrence of drought, these cells survive a combination of drought and high temperature to a better extent as their counterparts at temperate areas.

## Experimental procedures

### Strain, growth conditions and culture media

Fungal strains from the order Eurotiales (Table 3) were grown at 25–40°C on oatmeal agar (OA) or malt extract agar supplemented with 40% sucrose (MEA40S) (Samson and Houbraken, 2010). Inoculation was performed using a glycerol stock solution of conidia (10^6^ spores ml^−1^). *Neosartorya fischeri* (CBS 317.89) and *T. macrosporus* (CBS 580.72) cultures were routinely grown at 30°C on OA. Ascospores of these species were heat activated for 2 min at 85°C (Dijksterhuis and Samson, 2002; Dijksterhuis *et al*., 2002). Agar medium was inoculated by spreading 100 µl of a heat-activated suspension containing 10^7^ ascospores ml^−1^. After 40 days of growth, ascospores were harvested by collecting fungal material from cultures with a glass spatula. The mixture of hyphae and ascomata was transferred to 9 ml ice-cold 10 mM ACES buffer (pH 6.8) supplemented with 0.02% Tween-80 (Sigma-Aldrich, Zwijndrecht, the Netherlands), after which 1 cm^3^ of sterile glass beads (1:1 ratio of beads with a diameter of 0.10–0.11 mm and 1.0 mm) was added. Ascospores were released from cleistothecia by vortexing for 1–2 min and sonicating for 5 min using an ultrasonic cleaner 2510E-MT (Branson Ultrasonics Corporation, Danbury, CT, USA). Filtration through sterile glass wool removed the mycelial debris and remnants of the ascomata. The spores were washed three times with ice-cold ACES buffer, and centrifuged (5 min, 1100 *g*) after each washing step. If not immediately used for experiments, pellets of ascospores were stored in ACES buffer at −80°C.

### Monitoring heat resistance of ascospores

Ascospores were heated in solution (wet heat) or vacuum dried (dry heat). After the heat treatment, the germination percentage was measured with one of the following methods. Heat-treated spores were diluted to 10^4^ and 10^3^ spores ml^−1^. One hundred microlitre was spread on malt extract agar (MEA) plates and incubated for 2–3 days at 30°C. Germination percentage was based on the number of colony-forming units. Alternatively, heat-treated ascospores were inoculated on 1–2 mm thin slices of MEA (10^6^ spores ml^−1^), placed on an objective glass. The MEA slides were incubated for 14–16 h at 30°C in a water-saturated container, after which the germinated spores were counted by light microscopy (Zeiss Axioskop 2 plus microscope). A spore was considered to be germinated when a germ tube (initial) was visible. At least 100 spores were evaluated in triplicate.

#### Wet-heat treatment

Ascospores were suspended in hot ACES buffer (85°C). The ascospore suspension (10^6^ spores ml^−1^) was immediately transferred to an 85°C water bath and shaken at 150 r.p.m. The spore suspension was cooled after 0, 2, 10 or 30 min by adding ice-cold ACES buffer to a final concentration of 10^3^ spores ml^−1^, after which MEA plates were inoculated. Alternatively, the spore suspension was cooled on ice and 10 µl was used to inoculate an MEA slice positioned on an objective glass. Germination was determined as described above using microscopy.

#### Dry-heat treatment

Ascospores (10^7^ spores in 10 µl) were vacuum dried for 1 h (Savant SpeedVac DNA 110 Concentrator, Thermo Scientific, Erembodegem-Aalst, Belgium) in a 1.5 ml Eppendorf tube. The dry spores were kept for 7 days at ambient temperature (25°C) and ambient humidity (RH of 45–85%), called the ambient-dry treatment. Alternatively, spores were kept for 7 days at 25°C in a desiccator filled with silica with a RH of 0.5–2%, the so-called extreme dry condition. After incubation, the dried spores were heated at 25°C, 60°C, 70°C or 80°C for 1 h in a heat block. Subsequently, the ascospores were resuspended in ACES buffer (10^6^ spores ml^−1^) and heated at 85°C for 0–30 min at 150 r.p.m. in a water bath to evaluate heat activation and subsequent thermal inactivation as described above.

### Microviscosity determination with ESR spectroscopy

The cytoplasmic microviscosity of spores was determined by ESR spectroscopy as previously described (Dijksterhuis *et al*., 2007; Van Leeuwen *et al*., 2010). Perdeuterated TEMPONE (Sigma, St Louis, MO, USA) was used as a spin label. Potassium FC [K_3_Fe(CN)_6_] was used to quench the extracellular spin label signal. The final concentration of TEMPONE and FC in samples was 1 mM and 120 mM respectively. At these concentrations, the narrow line spectrum of TEMPONE originates exclusively from spin probe molecules in the cytoplasm, and therefore can be used to characterize cytoplasmic viscosity. The ESR spectra were recorded with an X-band 300E ESR spectrometer (Bruker Analytik, Rheinstetten, Germany).

The rotational correlation time (τ_c_) of TEMPONE in the cytoplasm of ascospores was calculated from the ESR spectra making use of the equation τ_c_ = KΔW_+1_(√h_+1_/h_−1_-1), where K is a constant (Kuznetsov *et al*., 1971) with a value of 6.5 10^−10^ s, ΔW_+1_ is the peak-to-peak width of the low-field (left-hand) line of the spectra (in gauss) and h_+1_, and h_−1_ are the heights of the low-field (left hand) and high-field (right hand) lines respectively (Kivelson, 1960). The cytoplasmic microviscosity was calculated from the rotational correlation time using the Stokes-Einstein relationship τ_c_ = 4π(a)^3^η/3kT, where a is the molecular radius of TEMPONE, η is the effective viscosity, k is the Boltzmann constant and T is the absolute temperature in Kelvin. The molecular radius of TEMPONE is usually defined as 3 Å (Keith and Snipes, 1974).

### Cell free extracts of ascospores

Ascospores were frozen in liquid nitrogen and transferred to a stainless steel grinding jar (Qiagen, Venlo, the Netherlands) cooled with liquid nitrogen and homogenized with the Qiagen Tissuelyser (2 min at 30 strokes s^−1^). 0.5–1 ml Milli-Q water was added and grinding was continued for an additional 2 min at 30 strokes s^−1^. Samples were thawed, transferred to a 2 ml Eppendorf tube and centrifuged at 4°C for 30 min at 10.000 *g*. The supernatant was heated for 30 min at 95°C to inactivate oligosaccharide-degrading enzymes and centrifuged again for 30 min at 10.000 *g*. The supernatant was filtered (0.2 µm acrodisc Cr 13 mm Syringe filter, Pall Life Science, Mijdrecht, the Netherlands) and stored at −80°C until used for further analysis.

### HPLC

The amount of glucose, trehalose, isobemisiose, neosartose, fischerose, glycerol and mannitol in cell-free extracts were determined using an HPLC (Waters, Etten-Leur, the Netherlands) equipped with a 2414 refractive index (RI) detector, a 515 HPLC pump, a pump control module II, a 717 plus autosampler and a cation-exchange column Sugar-Pak I. The mobile phase (0.1 mM Ca EDTA in milliQ-water) had a flow of 0.5 ml min^−1^. Sample volumes of 10 µl were run for 20 min using column and RI detector temperatures of 50°C. Peak integrations and calculations were performed by the Empower software (Waters). Retention time of the peaks was compared with those of 0.01–0.50% w/v trehalose, isobemisiose, neosartose, fischerose, mannitol, glucose and glycerol.

### TLC

Ascospore cell-free extracts and partially purified oligosaccharide preparations were spotted (2 µl) on TLC sheets (Merck Kieselgel 60 F254, 20 × 20 cm) and run using 2:1:1 n-butanol : acetic acid : water as the mobile phase. Sugar containing compounds were visualized by orcinol/sulfuric acid staining (100 mg orcinol monohydrate, 95 ml methanol, 5 ml sulfuric acid) using glucose, trehalose, raffinose, verbascose and stachyose as standards.

### Compatible solutes

The polyols mannitol and glycerol and the sugars glucose, trehalose, sucrose, verbascose and stachyose were ordered by Sigma-Aldrich. Isobemisiose, neosartose and fischerose were synthesized (Kuestner, Palumbo and Snyder, unpublished results). The first batch of isobemisiose used for nuclear magnetic resonance was acquired from Dr. T. Nishimoto and Dr. H. Watanabe of the Glycoscience Institute of Hayashibara Biochemical Laboratories.

### Phylogenetic analysis of ascospore producing species within the family Trichocomaceae

Genomic DNA was extracted from the mycelium of fungal strains (Table 3) that had been grown for 3–5 days on MEA agar plates using the UltraClean Microbial DNA Isolation kit (MO BIO Laboratories, USA). The ITS and LSU fragments were amplified and sequenced as described (Houbraken *et al*., 2007). The sequences were aligned with Muscle within the Mega 5.1 software package (Tamura *et al*., 2011). Genes were concatenated in SEAview (Galtier *et al*., 1996; Gouy *et al*., 2010). PAUP 4.10b (Swofford, 2002) was used to test compatibility between the two databases. The appropriate substitution model for the maximum likelihood phylogenetic analysis was calculated with Mega 5.1 using the T92 model (Tamura, 1992) with gamma distribution with invariant sites. Statistical support for branch nodes was calculated using a bootstrap analysis of a 1000 replicates. The *Talaromyces* clade was used as an outgroup.

### FTIR spectroscopy

Glasses were formed by drying 2.5 µl of a solution containing a total of 50 mg sugar and/or mannitol ml^−1^ on circular CaF_2_ windows (2−13 mm) for at least 1 week in a cabinet that was continuously purged with dry air at a RH of 3% at 24°C. Infrared absorption of the samples was measured using a Perkin–Elmer (Massachusetts, USA) series 1725 FTIR spectroscope equipped with an external beam facility to which a Perkin–Elmer IR microscope was attached. The microscope was equipped with a narrowband mercury–cadmium–telluride LN_2_ (liquid nitrogen)-cooled IR detector. The temperature was regulated by a computer-controlled device activating the LN_2_ pump, in conjugation with a power supply for heating the cell. The temperature of the sample was recorded separately using a PT-100 element that was located very close to the sample windows. The acquisition parameters were 4 cm^−1^ resolution, with 32 co-added interferograms, at a 3600–100 cm^−1^ wavenumber range.

Spectral analysis was described by Wolkers and colleagues (1998,2001) making use of the infrared Data Manager Analytical Software. The FTIR spectra were measured from −10°C to 140°C with a temperature increase of 1.5°C min^−1^, back to −10°C at a speed of 2°C min^−1^, and then to 140°C again with a temperature increase of 1.5°C min^−1^. The melting of glasses was monitored by the position of the band region between 3600 and 3000 cm^−1^ (OH stretching vibration, νOH) and the region between 1300 and 1000 cm^−1^ (OH bending vibration, δOH). The band position was calculated as the average of the spectral positions (*n* = 50) at 75% of the total peak height. The relation between the wave number of the OH stretching band as function of the temperature of the samples was visualized in a plot. The point of crossing between the regression lines in both solid-like and liquid regions of the plot was used to estimate T_g_ (Wolkers *et al*., 1998). The rate of change of the vibrational energy with temperature (cm^−1^ °C^−1^) is defined as the WTC and gives information on the average strength of hydrogen bonding between the compatible solutes (Wolkers *et al*., 1998; Kets *et al*., 2004).

## Notes

1 This said, under some conditions, fungal spores may preferentially accumulate glycerol, erythritol and/or arabitol (Hallsworth and Magan, 1994,1995; Hallsworth *et al*., 2003)

2 Examples include *Saccharomyces cerevisiae* and some extreme, prokaryotic halophiles (see also above): Hallsworth (1998); Hallsworth *et al*. (2007); Williams and Hallsworth (2009); Cray *et al*. (2013a); Lievens *et al*. (2014); Oren and Hallsworth (2014); and Yakimov *et al*. (2014)

## Supporting Information

Fig S1 Diameter and thickness of the cell wall of *T. macrosporus* (left) and *N. fischeri* (right) ascospores. The ascospore cell wall of *N. fischeri* was made visible by staining with 5-(and-6)-carboxyfluorescein. The ascospore cell wall of *T. macrosporus* is highly auto-fluorescent and no fluorescent dye was needed.
