# Supplementary figures and images for: Functionality and prevalence of trehalose-based oligosaccharides as novel compatible solutes in ascospores of Neosartorya fischeri (Aspergillus fischeri) and other fungi
Source: Environ Microbiol. 2014 Oct 22;17(2):395–411. doi: 10.1111/1462-2920.12558 (PMC4371660; doi:10.1111/1462-2920.12558)

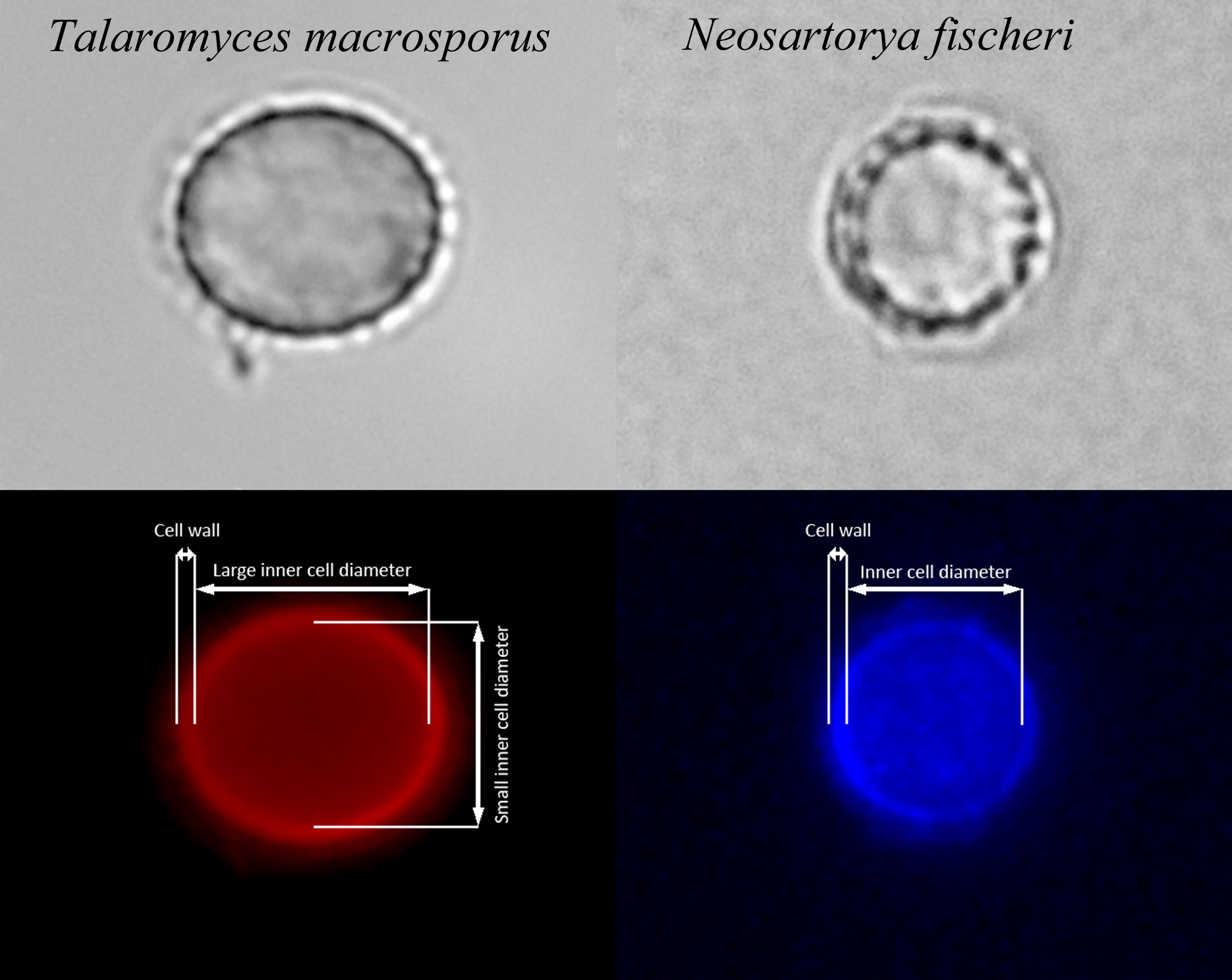

Supplement: Fig S1 — Diameter and thickness of the cell wall of T. macrosporus (left) and N. fischeri (right) ascospores. The ascospore cell wall of N. fischeri was made visible by staining with 5-(and-6)-carboxyfluorescein. The ascospore cell wall of T. macrosporus is highly auto-fluorescent and no fluorescent dye was needed. [file emi0017-0395-sd1.tif]
